# Melatonin suppresses fibrotic responses induced by cigarette smoke via downregulation of TGF-β1

**DOI:** 10.18632/oncotarget.21680

**Published:** 2017-10-09

**Authors:** Na-Rae Shin, Ji-Won Park, In-Chul Lee, Je-Won Ko, Sung-Hyeuk Park, Joong-Sun Kim, Jong-Choon Kim, Kyung-Seop Ahn, In-Sik Shin

**Affiliations:** ^1^ College of Veterinary Medicine (BK21 Plus Project Team), Chonnam National University, Gwangju 500-757, Republic of Korea; ^2^ Natural Medicine Research Center, Korea Research Institute of Bioscience and Biotechnology, Chungbuk 363-883, Republic of Korea; ^3^ Natural Product Research Center, Jeonbuk Branch, Korea Research Institute of Biosciences and Biotechnology, Jeongeup 580-185, Republic of Korea; ^4^ Research Center, Dongnam Institute of Radiological and Medical Science (DIRAMS), Busan 619-953, Republic of Korea

**Keywords:** melatonin, cigarette smoke, pulmonary fibrosis, TGF-β1, MAPK

## Abstract

Cigarette smoke (CS) is the most important risk factor in the development of chronic obstructive pulmonary disease (COPD). Pulmonary fibrosis is an irreversible response and important feature of COPD. In this study, we investigated the effects of melatonin on fibrotic response in development of COPD using a CS and lipopolysaccharide (LPS) induced COPD model and cigarette smoke condensate (CSC)-stimulated NCI-H292 cells, a human mucoepidermoid cell line. Mice were exposed to CS for 1 h per day (8 cigarettes per day) from day 1 to day 7 and were treated intranasally with LPS on day 4. Melatonin (10 or 20 mg/kg) was injected intraperitoneally 1 h before CS exposure. Melatonin decreased the inflammatory cell counts in bronchoalveolar lavage fluid (BALF), with a reduction in transforming growth factor (TGF)-β1. Melatonin inhibited the expression of TGF-β1, collagen I and SMAD3 phosphorylation in lung tissue exposed to CS and LPS. In CSC-stimulated H292 cells, melatonin suppressed the elevated expression of fibrotic mediators induced by CSC treatment. Melatonin reduced the expression of TGF-β1, collagen I, SMAD3 and p38 phosphorylation in CSC-stimulated H292 cells. In addition, cotreatment with melatonin and TGF-β1 inhibitors significantly limited fibrotic mediators, with greater reductions in the expression of TGF-β1, collagen I, SMAD3 and p38 phosphorylation than those of H292 cells treated with TGF-β1 inhibitor alone. Taken together, melatonin effectively inhibited fibrotic responses induced by CS and LPS exposure, which was related to the downregulation of TGF-β1. Therefore, our results suggest that melatonin may suppress the pulmonary fibrotic response induced by CS.

## INTRODUCTION

Cigarette smoke (CS) is considered an important risk factor for the deterioration of the normal respiratory structure and function. In particular, pulmonary fibrosis is an irreversible alteration and a primary cause of mortality in patients with pulmonary diseases such as chronic asthma and chronic obstructive pulmonary disease (COPD) [1]. During the development of pulmonary fibrosis, extracellular matrix (ECM) deposition, including collagen and fibronectin, is markedly increased via the release of various cytokines [2]. Among these cytokines, transforming growth factor (TGF)-β1 is closely associated with the progression of fibrotic changes [3]. TGF-β1 induces collagen deposition, increases ECM production, and increases α-smooth muscle actin (α-SMA) expression in lung tissue via phosphorylation of SMAD, which eventually causes pulmonary fibrosis [4–6]. A recent study demonstrated that cigarette smoke modulated the TGF-β1/SMAD pathway in COPD [7, 8]. In a clinical trial, alteration of TGF-β1/SMAD signaling was observed with pulmonary fibrosis in a patient with COPD [9]. Considering these findings, the regulation of TGF-β1/SMAD is an important strategy for controlling pulmonary fibrosis in COPD.

Melatonin is synthesized by various organs, including the pineal gland, and widely distributed in living organisms [10]. Recent studies have demonstrated that melatonin possesses additional properties including anti-oxidant, anti-cancer, anti-inflammatory, and immunomodulatory effects [11–17]. Moreover, the antioxidant effects of melatonin are not only because of an electron donor effect but also could be associated with the elevation of antioxidant enzymes, including superoxide dismutase, linked to reduced oxidative stress [18]. In particular, Yeung et al. [19] demonstrated that melatonin suppresses fibrotic markers such as PC1 and TGF-β in rats with chronic intermittent hypoxia-induced myocardial injury. In addition, melatonin was shown to inhibit nicotine-induced vasculopathy via blocking the activation of extracellular signal-regulated kinase (ERK) and TGF-β1 [20]. However, melatonin suppressed the phosphorylation of mitogen-activated protein kinases (MAPKs) induced by lipopolysaccharide (LPS) or cigarette smoke condensate (CSC) [21, 22]. These results indicate the close relationship between melatonin and the non-SMAD pathway in the fibrotic response. Considering these lines of evidence, melatonin is considered to suppress pulmonary fibrosis induced by CS exposure.

We investigated the effects of melatonin on CS- and lipopolysaccharide (LPS)-induced pulmonary fibrosis by measuring fibrotic mediators and performing histological analyses. In addition, we further explored the mechanism for the protective effects of melatonin on pulmonary fibrosis using CSC-treated H292 cells with a focus on its role in the modulation of TGF-β1.

## RESULTS

### Melatonin decreases pathophysiological alterations of bronchoalveoar lavage fluid (BALF) induced by CS and LPS exposure

The number of neutrophils in the CS + LPS mice markedly increased than that in the normal controls; however, melatonin level significantly decreased than that in the CS + LPS mice (Table 1). Total cell count in the melatonin-treated mice also markedly decreased than that in the CS + LPS mice. These reductions were greater in the mice treated with 20 mg/kg melatonin than in those treated with 10 mg/kg melatonin. In the CS + LPS mice, ROS production in BALF was significantly increased than that in the normal controls, whereas ROS production was markedly decreased in the melatonin-treated mice than that in the CS + LPS mice (Table 2). In addition, the melatonin-treated mice exhibited markedly reduced levels of IL-6 and TNF-α in BALF than those in the CS + LPS mice, consistent with the results of a previous study [22]. TGF-β1 levels in BALF induced by CS and LPS exposure were markedly decreased in the melatonin-treated mice, and the decrease in the mice treated with 20 mg/kg melatonin was larger than that in the mice treated with 10 mg/kg melatonin.

**Table 1:** Inflammatory cell count in BALF

Cells	NC	CS+LPS	ROF	Mel 10	Mel 20
Neutrophils	0.0 ± 0.00^a^	583.4 ± 65.39^##^	360.3 ± 40.58^**^	445.3 ± 59.07^**^	405.1 ± 63.71^**^
Other inflammatory cells	20.85 ± 5.48	50.7 ± 11.55^##^	49.2 ± 8.42	44.7 ± 11.95	32.3 ± 9.43^*^
Total cells	20.8 ± 5.48	634.1 ± 61.10^##^	409.6 ± 40.48^**^	490.0 ± 70.68^**^	437.4 ± 69.61^**^

Inflammatory cell counts were determined by counting cells on five squares of 400x magnification field of microscope in BALF of all animals (*n* = 6 per group). NC, normal control mice treated with PBS only; CS+LPS, cigarette smoke + LPS intranasal instillation; ROF, roflumilast (10 mg/kg) + cigarette smoke + LPS intranasal instillation; Mel 10 and 20, melatonin (10 mg/kg and 20 mg/kg) + cigarette smoke + LPS intranasal instillation.

^a^Values are expressed as means ± SD.

^##^*p* < 0.01 level compared with the normal control

^***^P < 0.05 and *P* < 0.01 levels compared with the CS+LPS group, respectively.

**Table 2:** The production of ROS and cytokines in BALF

Items	NC	CS+LPS	ROF	Mel 10	Mel 20
**ROS production (Fluorescense)**	2717 ± 749.7^a^	16540 ± 571.4^##^	15132 ± 980.4^*^	15122 ± 788.1^*^	14295 ± 348.8^**^
**BALF contents (mg/mL)**	0.10 ± 0.011	0.37 ± 0.034^##^	0.20 ± 0.033^**^	0.30 ± 0.029^**^	0.29 ± 0.025^**^
**IL-6 (pg/mL)**	21.4 ± 4.58	102.4 ± 16.55^##^	70.0 ± 11.66^**^	74.2 ± 7.38^**^	70.1 ± 11.68^**^
**TNF-α (pg/mL)**	31.1 ± 11.53	316.8 ± 49.75^##^	205.6 ± 65.13^**^	224.4 ± 43.31^*^	218.4 ± 24.5^*^
**TGF-β1 (pg/mL)**	11.62 ± 4.04	454.3 ± 58.44^##^	248.9 ± 24.61^**^	315.7 ± 37.13^**^	270.9 ± 52.07^**^

NC, normal control mice treated with PBS only; CS+LPS, cigarette smoke + LPS intranasal instillation; ROF, roflumilast (10 mg/kg) + cigarette smoke + LPS intranasal instillation; Mel 10 and 20, melatonin (10 mg/kg and 20 mg/kg) + cigarette smoke + LPS intranasal instillation.

^a^Values are expressed as means ± SD.

^##^*p* < 0.01 level compared with the normal control

^***^P < 0.05 and *P* < 0.01 levels compared with the CS+LPS group, respectively.

### Melatonin inhibits the inflammatory and fibrotic responses in the lung tissue induced by CS and LPS exposure

**Figure 1:**
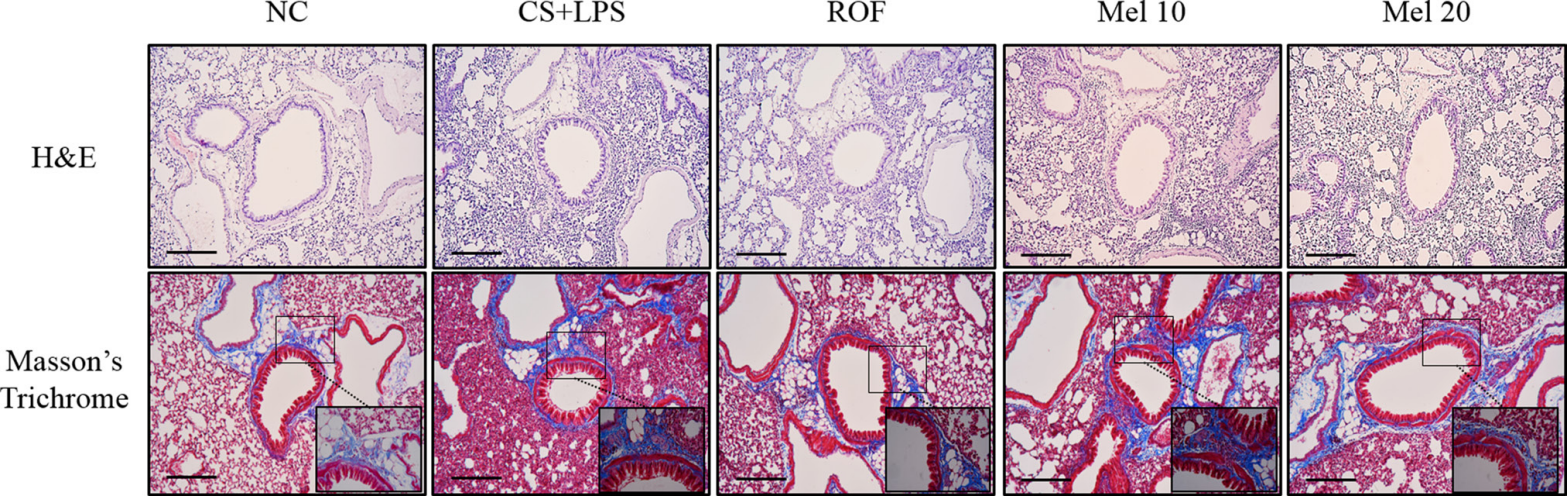
Melatonin attenuates inflammatory responses and collagen deposition in lung tissue induced by cigarette smoke and LPS exposure A representative figure of a peribronchial lesion in lung tissue stained with H&E and Masson's trichrome. NC, normal control mice treated with PBS only; CS + LPS, cigarette smoke + LPS intranasal instillation; ROF, roflumilast (10 mg/kg) + cigarette smoke + LPS intranasal instillation; Mel 10 and 20, melatonin (10 mg/kg and 20 mg/kg) + cigarette smoke + LPS intranasal instillation. Scale bars represent 200 μm.

**Figure 2:**
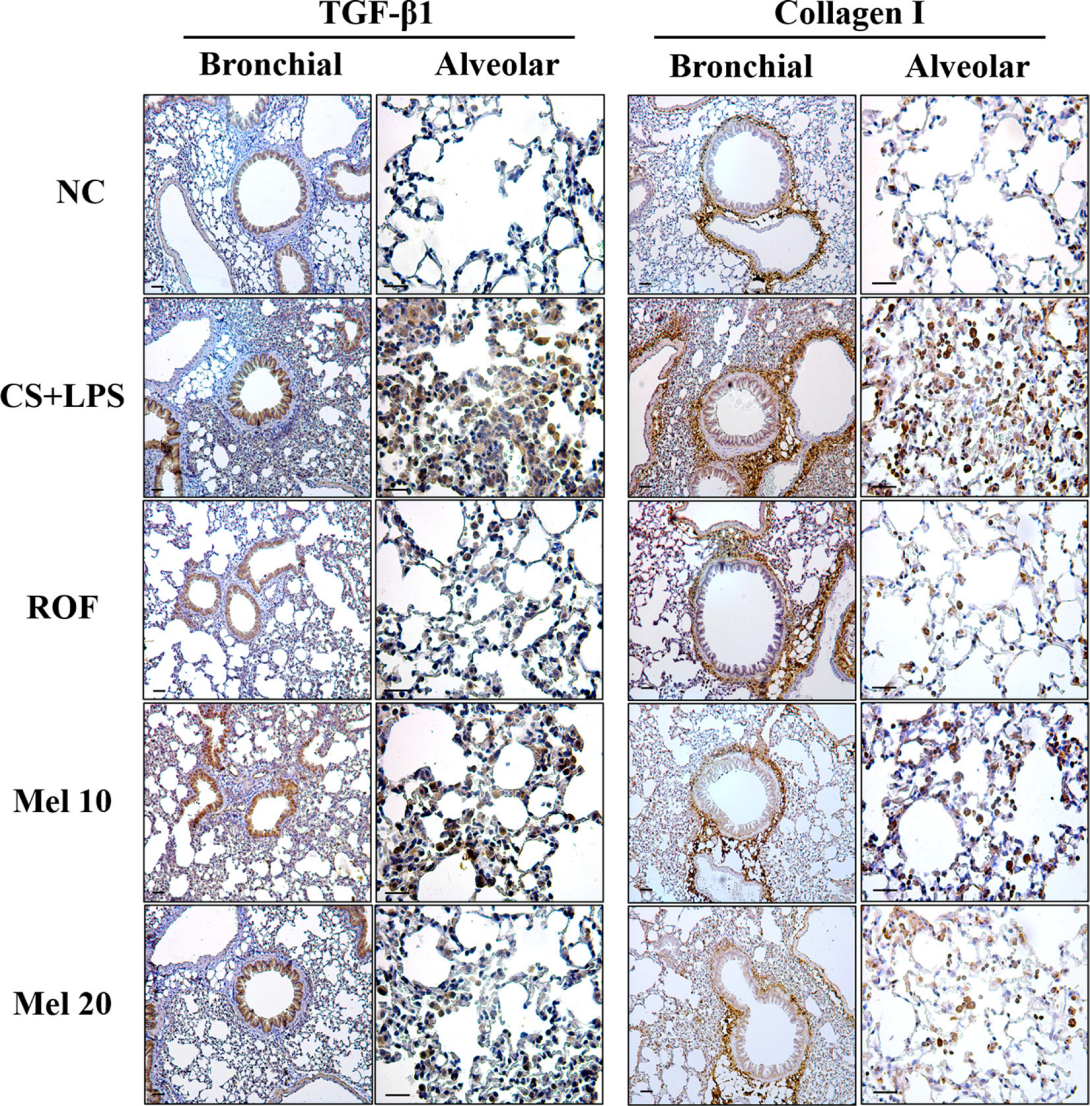
Melatonin reduces TGF-β1 and collagen I expression induced by cigarette smoke and LPS exposure in lung tissue (**A**) ROS production, (**B**) BALF contents, and (**C**) neutrophil elastase activity. NC, normal control mice treated with PBS only; CS + LPS, cigarette smoke + LPS intranasal instillation; ROF, roflumilast (10 mg/kg) + cigarette smoke + LPS intranasal instillation; Mel 10 and 20, melatonin (10 mg/kg and 20 mg/kg) + cigarette smoke + LPS intranasal instillation. Scale bars represent 50 μm.

In lung tissue stained with H&E, melatonin-treated mice exhibited obviously attenuated inflammatory responses in peribronchial and parenchymal lung lesions induced by CS and LPS exposure (Figure 1). In lung tissue stained with Masson's trichrome, CS and LPS exposed mice exhibited marked collagen deposition in lung tissue. However, melatonin-treated mice exhibited markedly decreased collagen deposition induced by CS and LPS exposure (Figure 2).

### Melatonin inhibits the expression of TGF-β1/SMAD in the lung tissue induced by CS and LPS exposure

**Figure 3:**
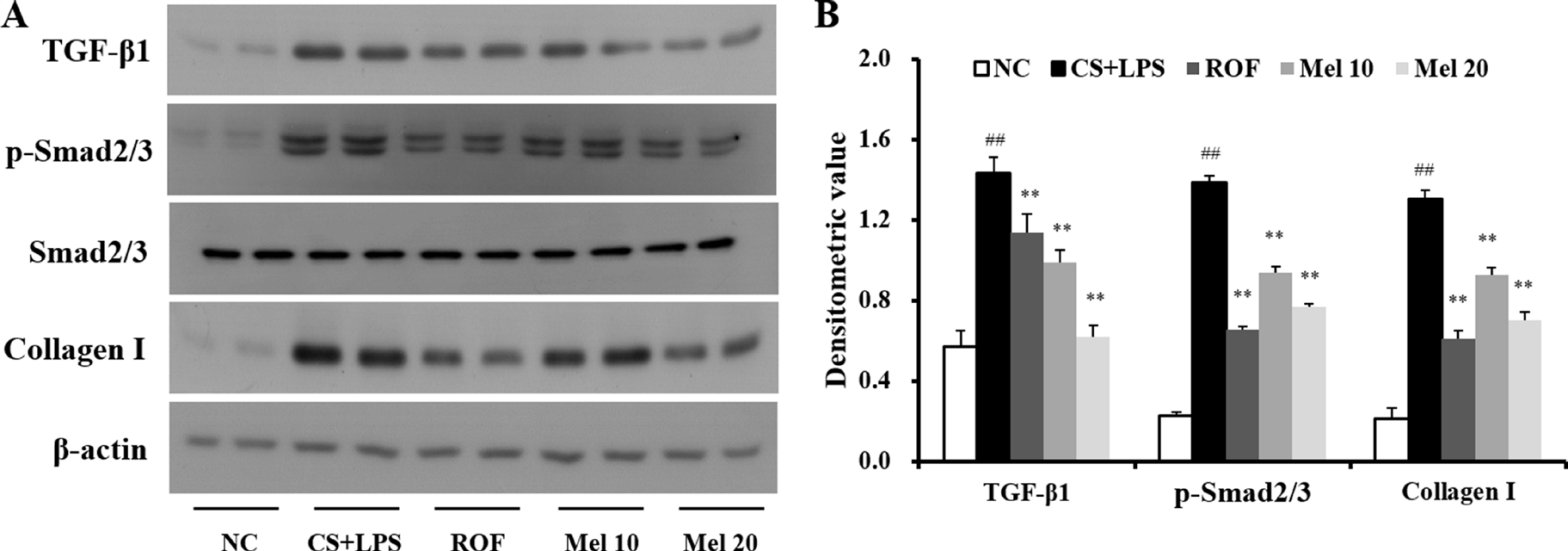
Melatonin reduces TGF-β1/SMAD3 signaling induced by cigarette smoke and LPS exposure (**A**) Gel showing protein expression and (**B**) the relative ratio of protein expression. NC, normal control mice treated with PBS only; CS + LPS, cigarette smoke + LPS intranasal instillation; ROF, roflumilast (10 mg/kg) + cigarette smoke + LPS intranasal instillation; Mel 10 and 20, melatonin (10 mg/kg and 20 mg/kg) + cigarette smoke + LPS intranasal instillation. ^##^Significantly different from the normal control group, *P* < 0.01; ^**^significantly different from the COPD group, *P* < 0.01.

In the CS + LPS mice, TGF-β1 and collagen I expressions in the lung tissue were increased than those in the normal controls; however, TGF-β1 and collagen I expressions in melatonin-treated mice were lower than those in CS + LPS mice. Regarding TGF-β1/SMAD3 signaling, the TGF-β1 expression and SMAD3 phosphorylation in lung tissue of the CS + LPS mice had increased more than those in the normal controls (Figure 3A). However, the TGF-β1 expression and SMAD3 phosphorylation in the melatonin-treated mice were significantly reduced than those in the CS + LPS mice. Moreover, the CS + LPS mice exhibited a marked increase in collagen I expression than the normal controls, whereas melatonin-treated mice showed a significant decrease than the CS + LPS mice (Figure 3B).

### Melatonin suppresses the expression of profibrotic mediators in CSC-stimulated H292 cells

**Figure 4:**
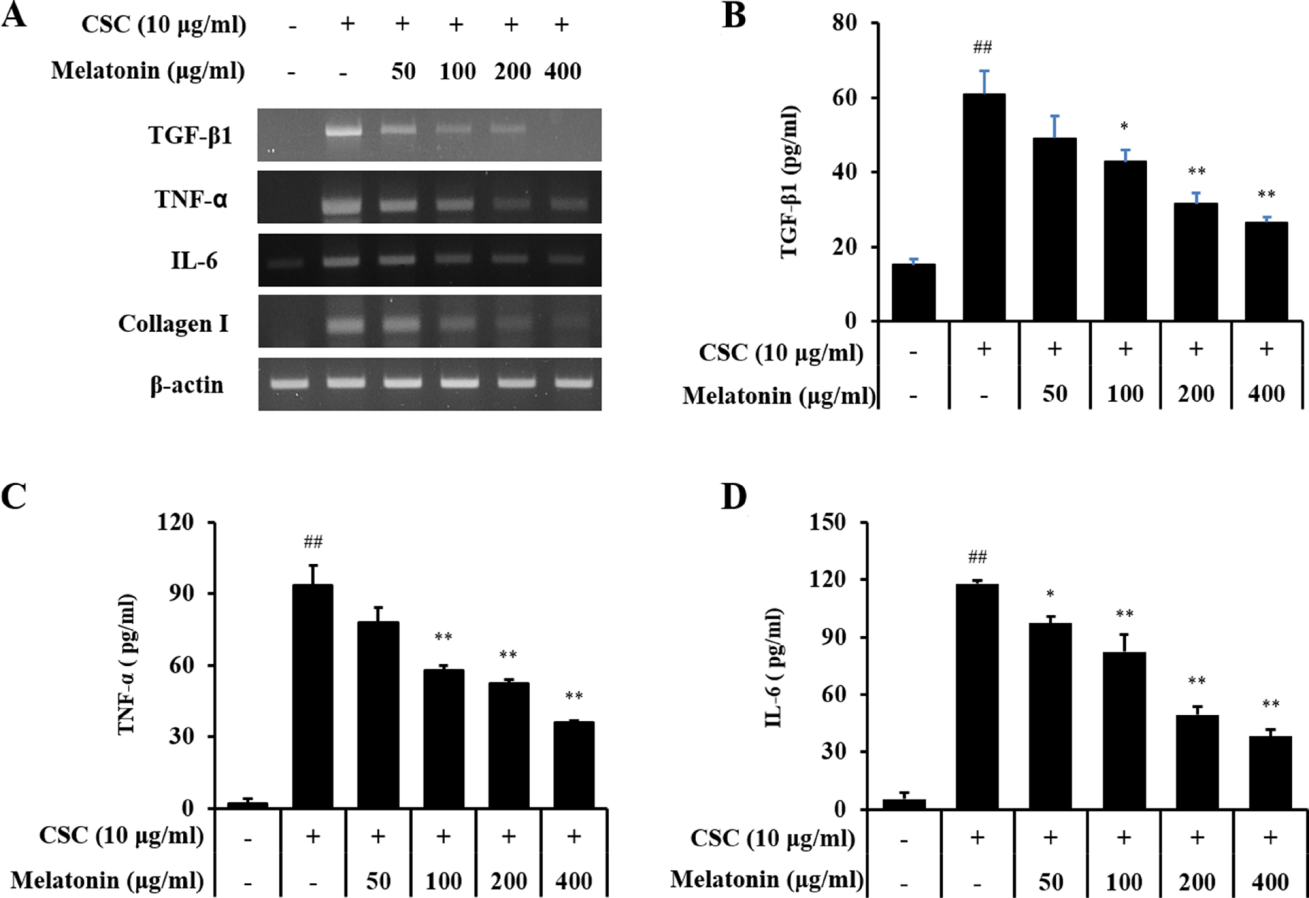
Melatonin inhibits the expression of TGF-β1, TNF-α, IL-6, and collagen I in H292 cells stimulated with cigarette smoke condensate (CSC) (**A**) mRNA expression of TGF-β1, TNF-α, IL-6, and collagen I, (**B**) TGF-β1 production, (**C**) TNF-α production, and (**D**) IL-6 production. ^##^Significantly different from non-stimulated H292 cells, *P* < 0.01; ^*^,^**^significantly different from CSC-stimulated H292 cells, *P* < 0.05 and *P* < 0.01.

In an *in vitro* experiment, CSC-stimulated H292 cells showed increased protein or mRNA levels of TGF-β1 along with an increase in IL-6 and TNF-α than the controls (Figure 4). However, melatonin-treated H292 cells exhibited significantly reduced increases in TGF-β1, IL-6, and TNF-α production stimulated by CSC.

### Melatonin reduces the expression of TGF-β1/SMAD in CSC-stimulated H292 cells

**Figure 5:**
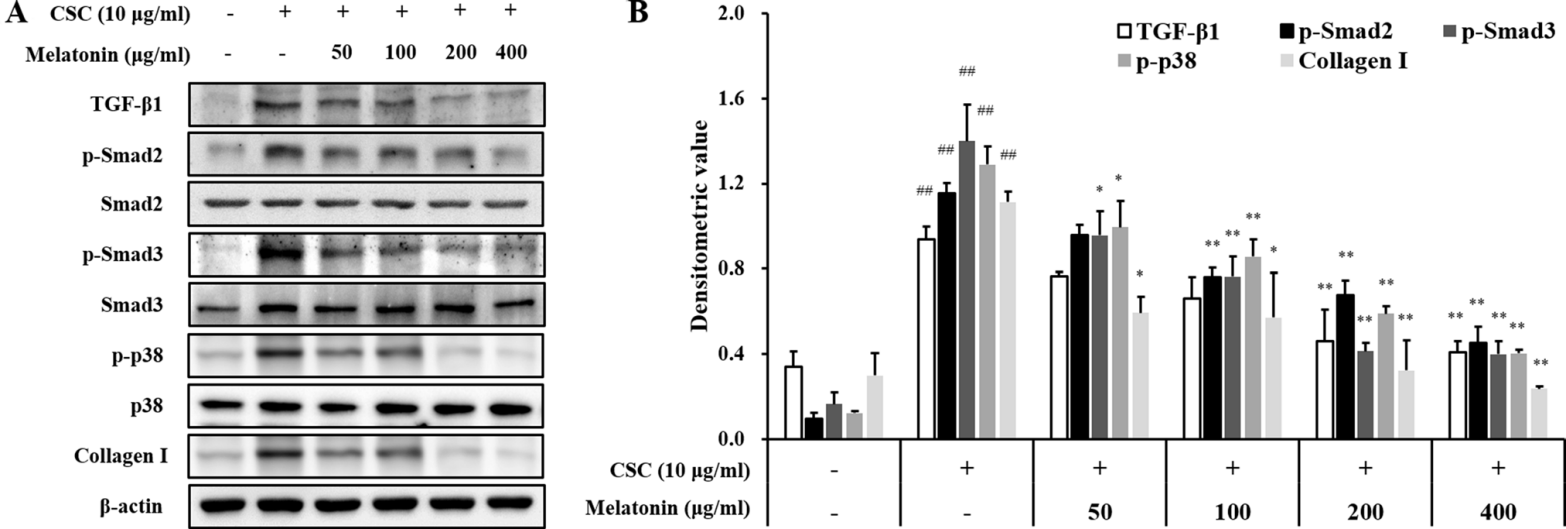
Melatonin decreases the expression of TGF-β1 and collagen I and phosphorylation of SMAD2/3 and p38 in H292 cells stimulated with cigarette smoke condensate (CSC) (**A**) Gel showing protein expression and (**B**) the relative ratio of protein expression. ^##^Significantly different from non-stimulated H292 cells, *P* < 0.01; ^*^,^**^significantly different from CSC-stimulated H292 cells, *P* < 0.05 and *P* < 0.01.

In addition, CSC-stimulated H292 cells showed increased TGF-β1 expression and phosphorylation of SMAD2/3 and p38 than the normal controls, whereas treatment with melatonin markedly reduced these increases in H292 cells induced by CSC stimulation than those in CSC-stimulated cells without melatonin (Figure 5). Collagen I expression was also markedly increased by CSC stimulation, which was significantly decreased by melatonin treatment.

### Melatonin decreases the fibrotic responses via downregulation of TGF-β1/SMAD in CSC-stimulated H292 cells

**Figure 6:**
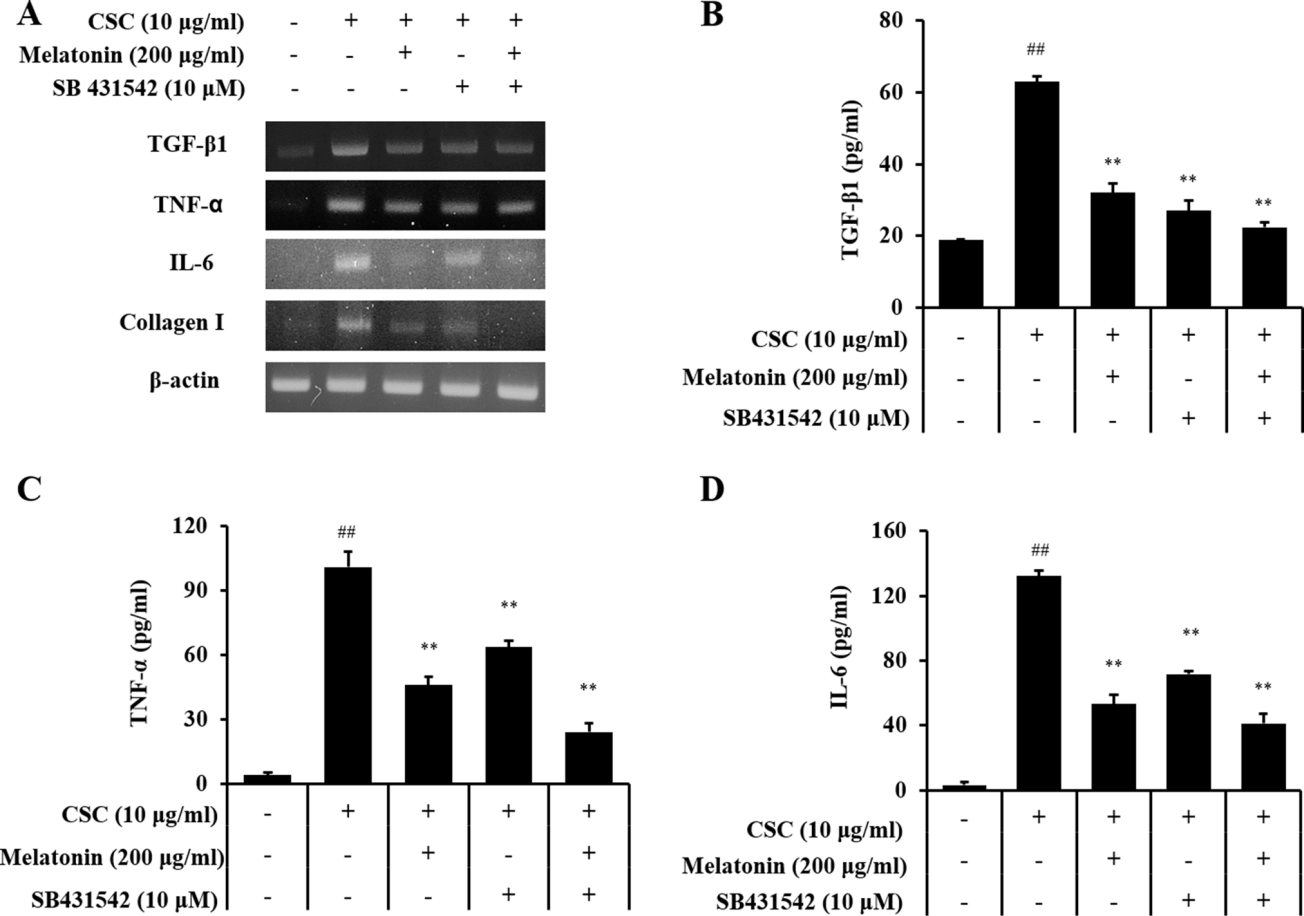
Melatonin suppresses the production of profibrotic mediators induced by CSC stimulation via down-regulation of TGF-β1 (**A**) mRNA expression of profibrotic mediators, (**B**) TGF-β1 production, (**C**) TNF-α production, and (**D**) IL-6 production. ^##^Significantly different from non-stimulated H292 cells, *P* < 0.01; ^**^significantly different from CSC-stimulated H292 cells, *P* < 0.01.

**Figure 7:**
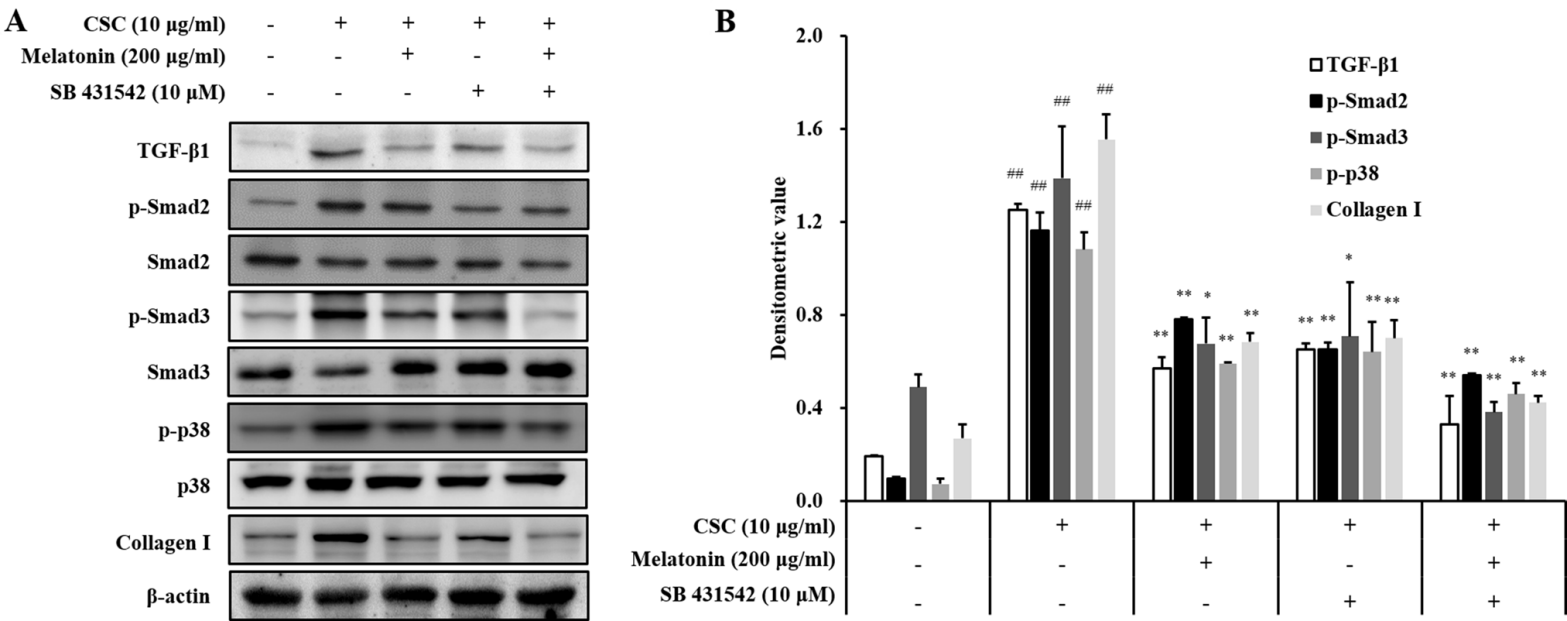
Melatonin down-regulates TGF-β1 in in H292 cells stimulated with cigarette smoke condensate (CSC) (**A**) Gel showing protein expression and (**B**) the relative ratio of protein expression. ^##^Significantly different from non-stimulated H292 cells, *P* < 0.01; ^*^,^**^significantly different from CSC-stimulated H292 cells, *P* < 0.05 and *P* < 0.01.

Treatment with a TGF-β1 inhibitor reduced the mRNA and protein expression of TGF-β1, IL-6, and TNF-α induced by CSC stimulation (Figure 6). Furthermore, TGF-β1, IL-6, and TNF-α expressions in cells cotreated with melatonin and the TGF-β1 inhibitor also reduced significantly than those in CSC-stimulated H292 cells, and this reduction was larger than that in the treatment with the TGF-β1 inhibitor alone. In addition, the TGF-β1 inhibitor treatment reduced collagen I expression and phosphorylation of SMAD3 and p38 induced by CSC stimulation in addition to the reduction in TGF-β1 expression (Figure 7). Furthermore, collagen I and phosphorylation of SMAD3 and p38 in cells cotreated with melatonin and the TGF-β1 inhibitor largely decreased than those treated with the TGF-β1 inhibitor alone.

## DISCUSSION

CS is an important risk factor in development of COPD. CS induces extensive airway inflammation via activation of inflammatory signaling and results in collagen deposition in lung tissues, resulting in pulmonary fibrosis. In the present study, we investigated the effects of melatonin on collagen deposition in CS and LPS exposed mice and CSC-stimulated H292 cells. Melatonin markedly decreased inflammatory cell counts and inflammatory mediators induced by CS and LPS exposure. Melatonin also significantly reduced fibrotic responses, including reductions in the TGF-β1/SMAD3 expression and collagen deposition in lung tissue. In an *in vitro* experiment, melatonin significantly reduced collagen I expression, which was accompanied by a decrease in TGF-β1/SMAD3 expression in CSC-stimulated H292 cells.

Melatonin significantly reduced the number of inflammatory cells in BALF from CS and LPS exposed mice. Melatonin effectively inhibited proinflammatory cytokines and ROS induced by CS and LPS, results that were also observed in the *in vitro* experiment. Melatonin suppressed the expression of proinflammatory mediators in CSC-stimulated H292 cells, consistent with the results of a previous study [22–25]. These anti-inflammatory effects of melatonin are closely related to the suppression of MAPK signaling reported in several studies [14, 26]. In particular, regarding MAPK signaling, the phosphorylation of ERK promotes fibrotic responses in damaged organs via the activation of various signaling molecules, including SMAD, AKT, and NF-κB [27]. Our previous study demonstrated that melatonin effectively suppressed inflammatory responses in CS-exposed mice and CSC-stimulated H292 cells by inhibiting ERK phosphorylation [28]. In the present study, melatonin reduced the collagen deposition in lung tissue in addition to the reductions in inflammatory mediators. Based on these results, the reduction in collagen deposition mediated by melatonin is likely related to its anti-inflammatory effects.

Pulmonary fibrosis is an irreversible response induced by various stimuli, resulting in the loss of normal lung function and death. Cigarette smoke is an important risk factor for the development of COPD and regarded as a key player in airway inflammation, emphysema, and pulmonary fibrosis in COPD patients. A recent study reported that cigarette smoke increases the epithelial to mesenchymal transition involved in the formation of peribronchiolar fibrosis [1]. COPD patients and smokers were found to have a significant increase in mesenchymal markers such as α-smooth muscle actin, vimentin, and collagen I than those in non-smokers [28]. Cigarette smoke extract-stimulated cells also showed an increase in the epithelial to mesenchymal transition [29]. In the present study, CS- and LPS-exposed mice exhibited increased collagen I expression than that in normal controls based histological evidence from lung tissue stained with Masson trichrome. However, melatonin-treated mice exhibited a marked decrease in collage I expression and collagen deposition (based on histological results) in lung tissue than those in CS- and LPS-exposed mice. These results were consistent with those from the *in vitro* experiments. CSC-stimulated H292 cells exhibited significantly increased collagen expression than that in the non-stimulated H292 cells, whereas melatonin-treated cells exhibited a marked decrease in collagen expression than that in CSC-stimulated H292 cells. These effects of melatonin agreed with the results of previous studies [30–32]. In particular, it was reported that melatonin reduces fibrotic responses by inhibiting the epithelial-mesenchymal transition in bleomycin-induced pulmonary fibrosis. These findings indicate that melatonin may suppress the fibrotic changes in lung tissue induced by cigarette smoke.

Various cytokines and growth factors are closely associated with pulmonary fibrosis. Among these mediators, TGF-β1 is thought to play a crucial role in the progression of fibrosis. TGF-β1 induces ECM production by pulmonary fibroblast and activates Smad signaling. In fibrosis, TGF-β1 binds to its receptor and then activates TGF-β1 receptor I-kinase, which induces the phosphorylation of Smad2 and Samd3. Phosphorylated Smad2 and Samd3 are from the Smad complex with Smad4, which translocates into the nucleus [33]. These events eventually produce fibrotic mediators such as α-SMA and collagens. A previous study demonstrated that cigarette smoke induces fibrotic responses via the activation of TGF-β1/Smad signaling [34]. These alterations have been observed in pulmonary fibroblast from patients with COPD [9, 35, 36]. In the present study, CS- and LPS-exposed mice exhibited activation of TGF-β1/SMAD signaling in addition to increased collagen I expression, results that were also confirmed in CSC-stimulated H292 cells. However, melatonin effectively suppressed the activation of TGF-β1/SMAD signaling, resulting in a reduction in collagen I expression in both *in vivo* and *in vitro* experiments. On the other hand, melatonin markedly suppressed the p38 phosphorylation induced by CSC. Similarly, we previously reported that melatonin reduces the ERK phosphorylation induced by CSC [22]. These results indicate that the effects of melatonin are associated with the down-regulation of the non-SMAD pathway in the fibrotic response. The protective effects of melatonin on the fibrotic response have been described in previous studies. Melatonin suppressed fibrotic responses on nicotine-induced vasculopathy via reductions in TGF-β1 [20]. Furthermore, melatonin attenuated fibrotic responses in the lung and kidney in a sepsis-induced injury model via suppression of TGF-β1/SMAD signaling [37, 38]. These documents were strongly supported the results of present study. Considering these evidence, melatonin has a potential to suppress pulmonary fibrosis induced by cigarette smoke via inhibition of TGF-β1/SMAD signaling.

In conclusion, melatonin markedly reduced the production of fibrotic mediators in cigarette smoke and LPS exposed mice and CSC-stimulated H292 cells. These properties were closely associated with the suppression of TGF-β1 expression. Therefore, our study suggest that melatonin may effectively suppress the pulmonary fibrotic response induced by cigarette smoke.

## MATERIALS AND METHODS

### COPD mouse model

Specific pathogen-free male C57BL/6N mice (6 weeks old, weight, 20–25 g) were purchased from Koatech Co. (Pyeongtaek, Republic of Korea) and used after 2 weeks of quarantine period and acclimatization. The mice were given sterilized tap water and standard rodent chow. All experimental procedures were approved by the Institutional Animal Care and Use Committee of Chonnam National University (Gwangju, Korea; CNU IACUC-YB-R-2015-20) and were performed in compliance with the Guidelines for Animal Experiments of Chonnam National University.

The procedure for the establishment of the animal model was based on our previous study [22]. Briefly, CS was generated from 3R4F research cigarettes (Kentucky reference cigarette, University of Kentucky, USA), and the mice received 1 h of CS exposure using a CS generator (Daehan Biolink, Incheon, Republic of Korea) from days 1 to 7. LPS was instilled intranasally on day 4 at a dose of 10 μg dissolved in 50 μL distilled water while the mice were under anesthesia (Zoletil 50^®^, Virbac Laboratories, Carros, France). The doses of melatonin (Sigma-Aldrich, Carlsbad, CA, USA) used (10 and 20 mg/kg) were based on a previous study [22], and it was administered to the mice for 7 days by intraperitoneal injection 1 h before cigarette smoke exposure. Roflumilast was used as a positive control drug; it is a phosphodiesterase-4 (PDE4) inhibitor and a recommended medication for treating COPD [39, 40]. Roflumilast was administered at dose of 10 mg/kg to mice for 7 days by oral gavage 1 h before cigarette smoke exposure. We used six animals per group in the animal experiment.

### Analysis of BALF

Twenty-four hours after the last CS exposure, BALF sampling was performed as described in a previous study [22]. To determine differential cell counts, BALF was stained with Diff-Quik® staining reagent (IMEB Inc., Deerfield, IL, USA), according to the manufacturer's instructions. Inflammatory cell counts were determined by average cell counting on five square of 400× magnification of microscope in BALF of all animals (*n* = 6 per group). Reactive oxygen species (ROS) in BALF was evaluated using 2′,7′-dichloroflurorescein diacetate (DCF-DA, Sigma-Aldrich) according to previous study [41]. The levels of proinflammatory mediators including interleukin (IL)-6 (Sensitivity: 15.6–1000 pg/mL, BD Biosciences, San Jose, CA, USA) and tumor necrosis factor (TNF)-α (Sensitivity: 15.6–1000 pg/mL, BD Biosciences) and TGF-β1 (Sensitivity: 8–1000 pg/mL, Invitrogen, Carlsbad, CA, USA) in the BALF were quantified by enzyme linked immunosorbent assay (ELISA), according to the manufacturer's protocols. The absorbance was measured at 450 nm using an ELISA reader (Molecular Devices, Sunnyvale, MA, USA).

### Histology and immunohistochemistry

The lung tissues were fixed in 10% (v/v) neutral buffered formalin. The tissues were embedded in paraffin, sectioned with a 4 μm thickness, and stained with hematoxylin and eosin (H&E) solution to estimate the inflammatory response. To evaluate pulmonary fibrosis, samples were stained with Masson's trichrome (Abcam, Cambridge, UK). To measure the protein expression, the sections were deparaffinized and hydrated, incubated in a solution of normal goat serum (Vector ABC Elite Kit; Vector Laboratories, Burlingame, CA, USA) for 60 min, reacted with rabbit anti-collagen I (1:200 dilution; Dako, Glostrup, Denmark) and –TGF-β1 (1:200 dilution; Abcam) overnight at 4°C, and washed with phosphate-buffered saline (PBS) containing 0.1% Tween-20. After washing, the bound primary antibodies were tagged with tetramethyl rhodamine isothiocyanate-labeled anti-rabbit IgG for 1.5 h at room temperature. After washing, the sections were counterstained, rinsed in PBS to terminate the reaction, and protected with cover slips for microscopic examination.

### Immunoblotting

Lung tissue was homogenized (1/10 w/v) using a homogenizer with a tissue lysis/extraction reagent (Sigma-Aldrich) containing a protease inhibitor cocktail (Sigma-Aldrich). The protein concentration of each sample was determined using the Bradford reagent (Bio-Rad Laboratories, Hercules, CA, USA) according to the manufacturer's instructions. Equal amounts of total cellular protein (30 μg) were resolved by 10% SDS-polyacrylamide gel electrophoresis and transferred to a poly-vinylidene fluoride membrane. The membrane was incubated with blocking solution (5% skim milk), followed by an overnight incubation at 4°C with the appropriate primary antibody. The following primary antibodies and dilutions were used: anti-β-actin (1:2000 dilution; Cell Signaling, Danvers, MA, USA), anti-TGF-β1 (1:1000 dilution; Abcam), anti-p-SMAD3 (1:1000 dilution; Abcam), anti-SMAD3 (1:1000 dilution; Abcam), and anti-collagen I (1:1000 dilution; Abcam). The blots were washed thrice with Tris-buffered saline containing Tween-20 (TBST) and then incubated with a 1:3000 dilution of a horseradish peroxidase (HRP)-conjugated secondary antibody (Jackson Immuno Research, West Grove, PA, USA) for 30 min at room temperature. The blots were again washed three times with TBST and then developed using an enhanced chemiluminescence kit (Thermo Scientific, San Diego, CA, USA). To determine relative ratio of protein expression, we analyzed the densitometric band values using ChemiDoc (Bio Rad Laboratories).

### Cell culture

NCI-H292 cells, a human airway epithelial cell line, were obtained from the American Type Culture Collection (ATCC, Manassas, VA, USA). The cells were maintained in RPMI 1640 supplemented with 10% fetal bovine serum (FBS) in the presence of penicillin (100 U/mL), streptomycin (100 μg/mL), and HEPES (25 mM) and incubated in a 5% CO_2_ incubator at 37°C. The cells were seeded on 96-well plates at a density of 5 × 10^4^ cells/well and incubated in serum-free medium in the presence various concentrations of melatonin. After incubation for 24 h, cell viability was evaluated via the 3-(4,5-dimethylthiazol-2-yl)-2,5-diphenyl tetrazolium bromide (MTT) assay. All the experiments were performed in triplicate.

### Preparation of cigarette smoke condensate (CSC)

CM6 (CORESTA approved Monitor No. 6) reference cigarettes were conditioned by ISO conditioning at a temperature of 22 ± 2°C and relative humidity of 60 ± 5% for 48 h or more. The reference cigarettes were smoked under ISO conditions (one puff/min, 35 mL puff volume over 2 seconds, every 60 seconds) using an automatic smoking machine (Borgwaldt RM20, Germany). CSC was trapped in a 92 mm Cambridge filter pad and then extracted with methanol for 3 days at room temperature. After extraction, the solvent was evaporated under vacuum and then stored at –80°C [42].

### Effects of melatonin on proinflammatory mediator and TGF-β1 production induced by CSC stimulation

The cells were seeded on 6-well plates at a density of 5×10^5^ cells/well, treated with a nontoxic concentration of melatonin, and incubated in the presence of cigarette smoke condensate (CSC, 10 μg/mL). To investigate the effects of melatonin on proinflammatory mediators and TGF-β1 expression, the cells were treated with melatonin (50, 100, 200, and 400 μM). The cells were harvested for 24 h after the melatonin treatment. Total RNA was isolated using TRIzol™ reagent (Invitrogen, Carlsbad, CA, USA) as instructed by the manufacturer, and a reverse transcription reaction was performed using a cDNA kit (Qiagen, Hilden, Germany) to investigate the effect of melatonin on the expression of *Tgf*-β1 mRNA. Polymerase chain reactions were performed using specific forward and reverse primers (*Tgf*-β, forward, 5′′- GGC GAT ACC TCA GCA ACC G, reverse, 5′- CTA AGG CGA AAG CCC TCA AT; *Il-6*, forward, 5′- GAC AGC CAC TCA CCT CTT CA-3′, reverse, 5′- AGT GCC TCT TTG CTG CTT TC-3′; *Tnf-α*, forward, 5′- TCA ACC TCC TCT CTG CCA AT -3′, reverse, 5′- CCT AAG CCC CCA ATT CTC TT -3′; *α-Sma*, forward, 5′- TGG GTG ACG AAG CAC AGA GC -3′, reverse, 5′- CTT CAG GGG CAA CAC GAA GC -3′; *β-Actin*, forward, 5′-CAT GTA CGT TGC TAT CCA GGC, reverse, 5′-CTC CTT AAT GTC ACG CAC GAT) and a premixed solution according to the manufacturer's instructions (Bioneer, Deajeon, Korea). TGF-β1 (Invitrogen), IL-6 (BD Biosciences) and TNF-α (BD Biosciences) protein levels were measured using commercial ELISA kit. The absorbance at 450 nm was measured using a microplate reader (Molecular Devices).

### Effects of melatonin on the expression of TGF-β1/SMAD signaling in CSC-stimulated H292 cells

The cells were treated with various concentrations of melatonin, followed by incubation in the presence of CSC (10 μg/mL) for 1 h or 24 h. The cells were collected via centrifugation, washed twice with PBS, and resuspended in an extraction lysis buffer (Sigma-Aldrich) containing protease inhibitors. The protein concentration was determined using a protein assay reagent (Bio-Rad Laboratories) according to the manufacturer's instructions. Western blotting was performed as described above, and the TGF-β1, collagen I, p-SMAD2, SMAD2, p-SMAD3, SMAD3, p-p38, and p38 expression levels were determined.

### Effects of melatonin on TGF-β1/SMAD signaling

To investigate the effects of melatonin on TGF-β1/SMAD signaling in CSC-stimulated cells, the cells were pretreated with melatonin (200 μM) and SB-431542 (TGF-β1 inhibitor, 10 μM, Millipore Co., Bedford, MA, USA) and incubated for 1 h or 24 h in presence of CSC (10 μg/mL). The proteins were extracted as described above. The TGF-β1, collagen I, p-SMAD2, SMAD2, p-SMAD3, SMAD3, p-p38, and p38 expression levels were investigated via western blotting.

### Statistical analysis

The data are expressed as the means ± standard deviation (SD). Statistical significance was determined using analysis of variance (ANOVA) followed by a multiple comparison test with Dunnett adjustment. A *P* value < 0.05 was considered significant.

## Abbreviations

α-SMA: α-smooth muscle actin
BALF: brochoalveolar lavage fluids
COPD: chronic obstructive pulmonary disease
CS: cigarette smoke
CSC: cigarette smoke condensate
DCF-DA: 2′,7′-dichloroflurorescein diacetate
ECM: extracellular matrix
ELISA: enzyme linked immunosorbent assay
ERK: extracellular signal-regulated kinase
FBS: fetal bovine serum
IL: interleukin
MTT: 3-(4,5-dimethylthiazol-2-yl)-2,5-diphenyl tetrazolium bromide
MAPKs: mitogen-activated protein kinases
LPS: lipopolysaccharide
PBS: phosphate buffered saline
PDE4: phosphodiesterase-4
TGF: transforming growth factor
TNF: tumor necrosis factor
